# A paraduodenal hernia revealed by bowel obstruction: case report and literature review

**DOI:** 10.11604/pamj.2018.31.120.13538

**Published:** 2018-10-17

**Authors:** Mustapha Ben Moussa, Ismail Nouhi, Taib Lachguar, Mohamed El Absi, El Hassan El Faricha El Alami, Mohamed El Ouanani, El Mahjoub Echarrab, Mohamed El Amraoui, Mohamed Rachid Chkof

**Affiliations:** 1 Surgical Emergency Department, Avicenna Hospital, Mohammed V University, Hay Souissi, Rabat, Morocco

**Keywords:** Internal hernia, intestinal obstruction, left paraduodenal hernia

## Abstract

Internal hernias are defined as the protrusion of abdominal viscera through an aperture in the intraperitoneal recesses, they are considered as a rare cause of intestinal obstruction. The paraduodenal hernias are the most common type of congenital hernia especially the left-sided ones. We report a case of a 46 year-old man presenting a left paraduodenal hernia with acute small bowel obstruction, which was firstly (preoperatively) assigned to a tumoral cause.

## Introduction

Internal hernias are defined by the protrusion of a viscus through a normal or abnormal peritoneal or mesenteric aperture within the confines of the peritoneal cavity [1]. They can be acquired following a surgical procedure or trauma and to congenital peritoneal defect [2]. They may remain silent, detected incidentally at laparotomy or at autopsy, or lead to intestinal obstruction. Paraduodenal hernias, the most common type, account for more than 50% of all cases and responsible for < 1% of small bowel obstruction [2, 3]. The left-sided PDH, more common, occurs generally in males [3] and has got 50% lifetime risk of developing small bowel obstruction and 20-50% rate of mortality [3]. We report the case of a 46 year-old man with signs of acute bowel obstruction diagnosed preoperatively to be due to a tumoral cause, but the emergency laparotomy revealed an obstructed left paraduodenal hernia (LPDH).

## Patient and observation

**Figure 1:**
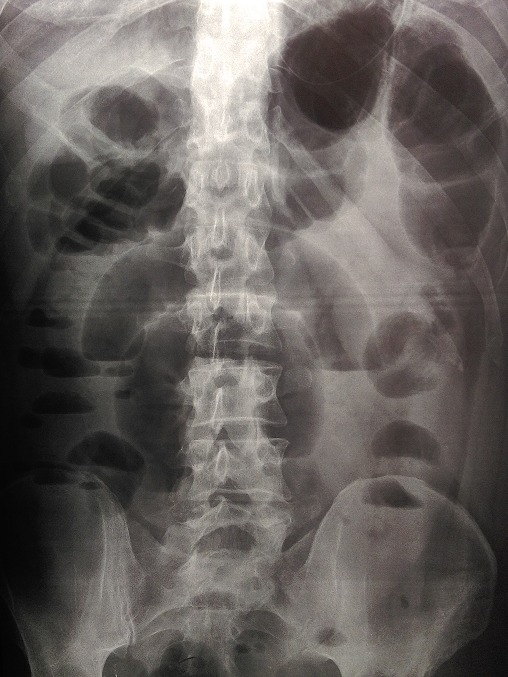
erect radiograph of the abdomen showing dilated small bowel loops with some air-fluid levels

**Figure 2:**
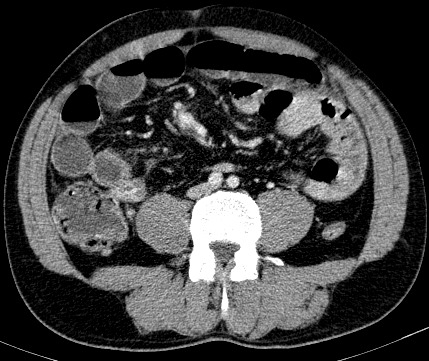
axial enhanced abdominal CT scan shows a cluster of jejunal loops in the Landzert’s fossa

A 46-year-old man was admitted to the emergency department with 3 days abdominal pain, colicky in nature, associated with vomiting and intestinal obstruction. On examination, the patient appeared in moderate pain, with mild tachycardia (85/mn), but normotensive and apyretic. Physical examination revealed a slight abdominal distension without any palpable mass. During digital rectal examination, the rectal ampulla was empty. Laboratory tests showed no noteworthy abnormalities except for leucocytosis of 16000 and a (pseudo) polycythemia (Hematocrit (HCT): the hematocrit = 54.1%. Hemoglobin (HGB) = 19.4g/dl. Plain abdominal X-ray in an erect position showed air-fluid levels of the small bowel loops in the upper abdomen (Figure 1). Abdominal CT scan was interpreted as an ileal obstruction associated with retractile mesenteritis around a hypervascularized pseudomass (Figure 2).

**Figure 3:**
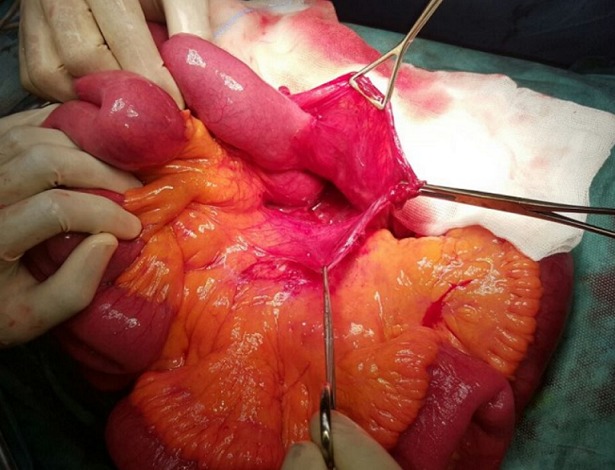
intra-operative view of the hernia sac. This image shows the Landzert’s fossa (empty), bounded by the first jejunal loop up and the neck of the sac around

**Figure 4:**
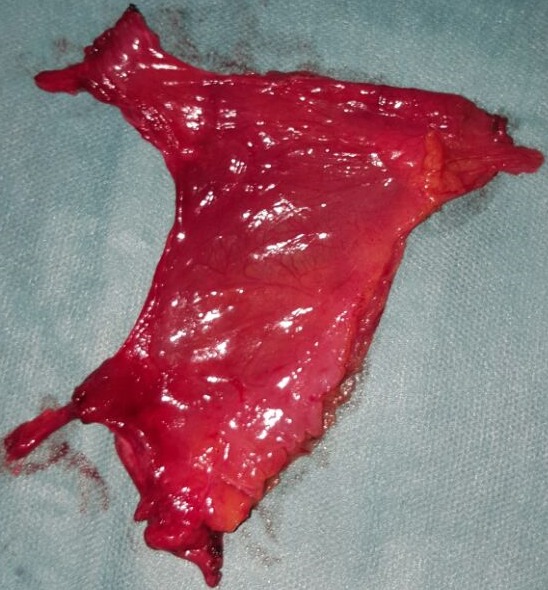
the hernia sac removed

The diagnosis of an acute bowel obstruction likely secondary to tumoral cause was made. Therefore, we decided to perform an emergency laparotomy which revealed that the proximal jejunum was prolapsed within a sac-like structure projected to the left of the midline (Figure 3). The root of mesentery was not twisted. The inferior mesenteric vessels were located in the medial edge of the anterior wall of the defect. Inside, the intestinal loops were viable and freely packed, but an adhesive band was noted stretched between the sac edge and ileocaecal region, across the last ileal loop causing its obstruction. Finally, the small bowel loops, found in viable condition, were easily reducible from the sac manually and intestinal resection was not necessary. The peritoneal sac was resected (Figure 4). No abdominal drain was placed. The postoperative recovery was uneventful: the patient had active bowel sounds on the first postop day and resumed oral intake on day 2. He was discharged on the fourth day.

## Discussion

Internal hernias are considered as an unusual cause of small-bowel obstruction, with a reported incidence of less than 2% [4]. Although Paraduodenal hernias, type of congenital hernias, are rare circumstance (0.2 - 0.9%), they account for 53% of all internal hernias [2]. The LPDH is 3 times more common than its right homologue [3]. Usually, males are more affected than females (3:1) and the fourth-sixth decade of life are the most involved [5, 6]. A literature search between 1980 and 2012 using PubMed revealed only 45 case reports. Median age at presentation was 47 (range of 18 - 82 years old) with male to female ratio of 3:1 [7].

Left paraduodenal hernias are congenital anomalies that arise during the rotation of the digestive tract, when the small bowel invaginates in an avascular segment of transverse descending mesocolon, localizing into a fossa (Landzert’s fossa) to the left of the fourth part of the duodenum, posterior to the inferior mesenteric vein and to left branches of the middle colic artery [5, 6, 8-10]. This congenital defect, potential space behind the mesocolon, is a consequence of fusion failure of mesocolon and the body wall peritoneum [11]. At autopsy, the Landzert’s fossa was found in about 2% of the population [9, 10]. However, in a recent study paraduodenal recess was found in 12% of cases [12].

Small bowel loops (usually jujenum) prolapse posteroinferiorly through the fossa into the left portion of the transverse mesocolon leading to its entrapment within this mesenteric sac [1, 13]. With a varied and non-specific clinical picture, paraduodenal hernias can be completely asymptomatic throughout life, or present with recurrent upper abdominal pain (43%) or with symptoms and signs of small bowel obstruction. Only in a third of cases, can left paraduodenal hernia lead to the appearance of a palpable abdominal mass in the left upper quadrant and a relaxation of the eccentric ileal loops at the headquarters of the same [5]. Many of the paraduodenal hernia can be diagnosed incidentally at laparotomy, autopsy or during radiological investigation for an unrelated disease [14]. The preoperative diagnosis of asymptomatic paraduodenal hernia is difficult, so imaging can be of no help if it is not done during symptomatic episode [3]. Among 45 recently reported cases of symptomatic left paraduodenal hernias, 19 cases (43%) were diagnosed before surgery [7]. Plain X-ray (first-line imaging exam in emergency department) may show signs of bowel obstruction, a mass effect with displacement of other abdominal organs by herniated bowel segment [4].

Abdominal CT scan is the standard for the diagnosis of the left paraduodenal hernia. It may show different typical radiological aspects related to hernia: a “cluster” of small bowel loops, an encapsulated saclike mass at level of the ligament of Treitz, a depression of the duodenal-jejunal junction, a mass effect on the rear wall of the stomach, congestion and overcrowding of the mesenteric vessels with frequent right displacement of the main mesenteric trunk and anterior upwards displacement of the inferior mesenteric vein, which delimits the hernial defect and a depression of the transverse colon [5, 9, 15]. Angiography may be helpful in demonstrating displacement or twisting of blood vessels [4].

Diagnostic laparoscopy for verification of diagnosis and simultaneous surgical intervention can be tried in cases that cannot be diagnosed with radiological method [16]. Surgery is always indicated (necessary), even in asymptomatic cases, because of the increased risk for a life-long incarcerated or strangulated hernia, which represent acute complications related to a 20 - 50% mortality rate [5]. Treatment is based on manual reduction of bowel loops with surgical repair of the abnormal defect: closure of hernial defect with continuous or interrupted suture, enlargement of defect or resection of the sac [17].

## Conclusion

Left paraduodenal hernia is an uncommon cause of small bowel obstruction, does not have distinctive clinical features therefore, it should be considered in a person with recurrent abdominal pain and intermittent bowel obstruction with no history of previous abdominal surgery. With a high lifetime risk of obstruction and high mortality rate, the diagnosis should occur preoperatively taking advantage of the important role of the modern imaging technique. The treatment is considered mandatory even in uncomplicated cases.

## Competing interests

The authors declare no conflict of interests.

## Authors’ contributions

Mustapha Ben Moussa: study design, data collection, statistical analysis, data interpretation, manuscript preparation, literature search, and writing. The other authors: study design, data collection and interpretation. All authors have read and agreed to the final manuscript.

## References

[1] Martin LC, Merkle EM, Thompson WM (2006). Review of internal hernias: radiographic and clinical findings. American Journal of Roentgenology.

[2] Selçuk D, Kantarci F, Ogüt G, Korman U (2005). Radiological evaluation of internal abdominal hernias. Turk J Gastroenterol.

[3] Virich G, Davies W (2010). A massive left paraduodenal fossa hernia as an unusual cause of small bowel obstruction. Ann R Coll Surg Engl.

[4] Blachar A, Federle MP, Dodson SF (2001). Internal hernia: clinical and imaging findings in 17 patients with emphasis on CT criteria. Radiology.

[5] Assenza M, Rossi D, Rossi G, Reale C, Simonelli L, Romeo V (2014). Laparoscopic management of left paraduodenal hernia. Case report and review of literature. II Giornale di Chirurgia.

[6] Palanivelu C, Rangarajan M, Jategaonkar PA, Anand NV, Senthilkumar K (2008). Laparoscopic management of paraduodenal hernia: mesh and mesh-less repairs. A report of four cases. Hernia.

[7] Al-Khyatt W, Aggarwal S, Birchall J, Rowlands TE (2013). Acute intestinal obstruction secondary to left paraduodenal hernia: a case report and literature review. World Journal of Emergency Surgery.

[8] Moon CH, Chung MH, Lin KM (2006). Diagnostic laparoscopy and laparoscopic repair of a left paraduodenal hernia can shorten hospital stay. JSLS.

[9] Khalaileh A, Schlager A, Bala M, Abu-Gazala S, Elazary R, Rivkind AI (2010). Left laparoscopic paraduodenal hernia repair. Surgical Endoscopy.

[10] Hassani KIM, Aggouri Y, Laalim SA, Toughrai I, Mazaz K (2014). Left paraduodenal hernia: A rare cause of acute abdomen. The Pan African Medical Journal.

[11] Bhuyan K, Hemanth G (2014). Left paraduodenal hernia causing acute bowel obstruction: report of a case and review of literature. International Surgery Journal.

[12] Tambe SV, Rana KK, Kakar A, Aggarwal S, Aggrawal A, Kakar S (2017). Clinical importance of duodenal recesses with special reference to internal hernias. Arch Med Sci.

[13] Armstrong O (2007). Internal hernias: anatomical basis and clinical relevance. Surg Radiol Anat.

[14] Kiyotaka Kurachi, Toshio Nakamura, Tadataka Hayashi, Yosuke Asai, Takayuki Kashiwabara, Akihito Nakajima, Shohachi Suzuki HK (2006). Left paraduodenal hernia in an adult complicated by ascending colon cancer: a case report. World J Gastroenterol.

[15] Uchiyama S, Imamura N, Hidaka H, Maehara N, Nagaike K, Ikenaga N, Hotokezaka M, Chijiiwa K (2009). An unusual variant of a left paraduodenal hernia diagnosed and treated by laparoscopic surgery: report of a case. Surg Today.

[16] Akbulut S (2012). Unusual cause of intestinal obstruction: left paraduodenal hernia. Case Rep Med.

[17] Zizzo M, Smerieri N, Barbieri I, Lanaia A, Bonilauri S (2016). Laparoscopic treatment of acute small bowel obstruction due to left paraduodenal hernia: a case report and literature review. Int J Surg Case Rep.

